# Identification and characterization of two CD4 alleles in Microminipigs

**DOI:** 10.1186/s12917-016-0856-8

**Published:** 2016-10-07

**Authors:** Tatsuya Matsubara, Naohito Nishii, Satoshi Takashima, Masaki Takasu, Noriaki Imaeda, Kayo Aiki-Oshimo, Kazuaki Yamazoe, Michinori Kakisaka, Shin-nosuke Takeshima, Yoko Aida, Yoshie Kametani, Jerzy K. Kulski, Asako Ando, Hitoshi Kitagawa

**Affiliations:** 1 United Graduate School of Veterinary Sciences, Gifu University, Gifu, 501-1193 Japan; 2 Department of Veterinary Medicine, Faculty of Applied Biological Sciences, Gifu University, 1-1 Yanagido, Gifu, 501-1193 Japan; 3 Viral Infectious Diseases Unit, RIKEN, Wako, 351-0198 Japan; 4 Department of Molecular Life Science, Division of Basic Medical Science and Molecular Medicine, Tokai University School of Medicine, Isehara, 259-1193 Japan; 5 School of Psychiatry and Clinical Neurosciences, The University of Western Australia, Crawley, WA 6009 Australia

**Keywords:** CD4 polymorphism, PCR-RFLP, Amino-acid substitution, Microminipigs

## Abstract

**Background:**

We previously identified two phenotypes of CD4+ cells with and without reactions to anti-pig CD4 monoclonal antibodies by flow cytometry in a herd of Microminipigs. In this study, we analyzed the coding sequences of CD4 and certified the expression of CD4 molecules in order to identify the genetic sequence variants responsible for the positive and negative PBMCs reactivity to anti-pig CD4 monoclonal antibodies.

**Results:**

We identified two CD4 alleles, *CD4.A* and *CD4.B*, corresponding to antibody positive and negative, respectively, by nucleotide sequencing of PCR products using CD4 specific primer pairs. In comparison with the swine CD4 amino-acid sequence [GenBank: NP_001001908], CD4.A had seven amino-acid substitutions and CD4.B had 15 amino-acid substitutions. The amino-acid sequences within domain 1 of CD4.B were identical to the swine CD4.2 [GenBank: CAA46584] sequence that had been reported previously to be a modified CD4 molecule that had lost reactivity with an anti-pig CD4 antibody in NIH miniature pigs. Homozygous and heterozygous *CD4.A* and *CD4.B* alleles in the Microminipigs herd were characterised by using the RFLP technique with the restriction endonuclease, *Bse*RI. The anti-pig CD4 antibody recognized pig PBMCs with *CD4.AA* and *CD4.AB*, but did not recognized those with *CD4.BB*. We transfected HeLa cells with the FLAG-tagged CD4.A or CD4.B vectors, and certified that transfected HeLa cells expressed FLAG in both vectors. The failure of cells to react with anti-CD4 antibodies in CD4.B pigs was associated to ten amino-acid substitutions in domain 1 and/or one amino-acid substitution in joining region 3 of CD4.B. We also found exon 8 was defective in some *CD4.A* and *CD4.B* resulting in the loss of the transmembrane domain, which implies that these CD4 proteins are secreted from helper T cells into the circulation.

**Conclusions:**

We identified that amino-acids substitutions of domain 1 in CD4.B gave rise to the failure of some CD4 expressing cells to react with particular anti-pig CD4 monoclonal antibodies. In addition, we developed a PCR-RFLP method that enabled us to simply identify the CD4 sequence variant and the positive and negative PBMCs reactivity to our anti-pig CD4 monoclonal antibodies without the need to use flow cytometric analysis.

**Electronic supplementary material:**

The online version of this article (doi:10.1186/s12917-016-0856-8) contains supplementary material, which is available to authorized users.

## Background

The CD4 molecule is a cell-surface glycoprotein receptor expressed by helper T cells, monocytes, macrophages, and dendritic cells; and its structure consists of four immunoglobulin-like domains (D 1 to D 4) as part of the extracellular domain, a transmembrane domain, and a cytoplasmic tail [1, 2]. The extracellular domain binds to the monomorphic region of MHC class II to increase the affinity of the T cell receptor to the antigen peptide-MHC class II complex [3, 4]. The cytoplasmic portion of CD4+ recruits tyrosine kinase, Lck, and the kinase enhances signal transduction in T cell activation [5, 6].

Microminipigs are extra-small and novel miniature pigs developed for biomedical research in Japan [7]. Recently, swine leukocyte antigen (SLA) haplotypes were assigned in a herd of Microminipigs in order to further investigate their immunological characteristics [8] during disease and infections. In the process of analyzing helper T cell function, we found that some pigs had CD4+ cells that could not be detected by flow cytometry while using three anti-pig CD4 monoclonal antibodies of the clones 74-12-4, MIL17, and PT90A [9]. The pedigree analysis indicated that the CD4-undetectable trait might be recessive, suggesting gene variation [9]. Failure of CD4 cells to react with an anti-pig CD4 antibody was reported previously in the NIH miniature swine [10] and the presence of partial nucleotide sequences and 10 amino-acid substitutions in exon 3 and 4 of two kinds of CD4 alleles (*CD4.1*, *CD4.2*) in these miniature swine might be the cause of helper T cells not reacting with the anti-pig CD4 antibody [11]. On the other hand, because Microminipigs have no consanguinity with NIH miniature swine, the cause for the failure of CD4 cells to react with the anti-pig CD4 antibody in Microminipigs might be different from NIH miniature swine. Hence, we need to clarify the variations in the CD4 nucleotide and amino acid sequences for the positive and negative antibody phenotypes in Microminipigs.

In this study, in order to clarify the reasons for the failure of the anti-pig CD4 antibodies to react with and detect peripheral CD4+ cells and to assess whether sequence variations within the CD4 molecules of Microminipigs might cause immunological alterations, we 1) sequenced and analyzed the coding sequence (CDS) of CD4 using genomic DNA and reverse transcribed (RT)-PCR products of CD4 mRNA in Microminipigs,, 2) developed a simple PCR-RFLP method to identify the CD4 sequence variant and the positive and negative PBMCs reactivity to anti-pig CD4 monoclonal antibodies, and 3) examined the expression of the CD4 alleles transfected into HeLa cells.

## Methods

### Microminipigs

Microminipigs were raised in a conventional environment at Fuji Micra Inc. (Fujinomiya, Japan) or Gifu University. This study was carried out along the Gifu University Laboratory Animal Guidelines.

### Flow cytometric analysis

Flow cytometry was performed as previously [9]. Briefly, peripheral blood mononuclear cells (PBMCs) of 231 Microminpigs were isolated using Lymphoprep (Axis Shield, Oslo, Norway), stained with a FITC-conjugated anti-pig CD4a antibody (clone 74-12-4, BD Biosciences, San Jose, CA), and analyzed using FACSCalibur (BD Biosciences) to classify the pigs with and without CD4 affinity for the 74-12-4 antibody. The data was analyzed with FlowJo version 7.6.5 software (FlowJo, Ashland, OR). The antibody reactivity with the CD4 protein was measured as the MFI (median of fluorescence intensity) of CD4+ cells in PBMCs.

### Direct sequencing of CD4 coding region

Genomic DNA was extracted from peripheral blood, pieces of tail or ear tissues of 11 Microminipigs (reactive CD4: six pigs, non-reactive CD4: five pigs) using a Wizard Genomic DNA Purification Kit (Promega Corporation, Madison, WI). CD4 gene-specific primer pairs for amplification and sequencing of the coding region (exons 2 to 10) were designed as shown in Table 1 based on a swine reference sequence [Genbank: NC_010447]. The PCR cycling parameters included an initial denaturation step of 4 min at 94 °C, 35 cycles consisting of 30 s at 94 °C, 30 s at 60 °C, 30 s at 72 °C, and a final extension step of 7 min at 72 °C. Sequencing was performed with ABI PRISM 3100 (Life Technologies, Carlsbad, CA) using Big Dye Terminator ver. 3.1 (Life Technologies). The nucleotide and deduced amino-acid sequence results were aligned using GENETYX version 10 (GENETYX, Tokyo, Japan) with three swine CD4 sequences: [GenBank: NM_001001908, GenBank: X65629 (*CD4.1*), GenBank: X65630 (*CD4.2*)].

**Table 1:** CD4 exonic primer sets for DNA sequencing

Amplification exon	Forward primer sequence	Reverse primer sequence	Length of PCR product size (bp)
2	5'-GTACCTGTGGGTGTCAGTTTAGAG-3'	5' -CTTACCCAGCACCAGATATTTTTC-3'	383
3	5'-CTCAGACTCAAACTGGGATGATTG -3'	5' -GATCCCAGAGTTTACTAGGAGCTG-3'	366
4	5'-AATGAGCAACTCAGATCAGAAGAGT-3'	5'-CTTATCCATCTCTGGACGGTTG-3'	363
5	5'-CTTCTCCTTGGGGATAGTGCAT-3'	5'-ACACTACAGCCACGAGCAGAG-3'	358
6	5'-GCCTAGAGCTAGATGGGAATTTAAG -3'	5'-GATTCCAGCCTCAGTTCAAACC-3'	617
7	5'-CTTTAGAGCAGACAAGTGCTAGGAA- 3'	5'-ACCATACCCATAACCCACTGACTC-3'	373
8	5'-AGCATAAGGATCAGACCCAAGTGT-3'	5'-TAACTCTGTGGCTTCTTGTCTCTC-3'	400
9	5'-GTTAATTCTGGGACAGATGGCTTC-3'	5'-CTCTCTTCACCCCTCCTCTTTG-3'	238
10-1	5'-CCATCTCTGTGCAGGAAAAGTC-3'	5'-AGCTGAGCTGCTTGGGTGATA-3'	698
10-2	5'-ACTGACGGAGCCACAGACTC-3'	5'-GGCTATCAACTTTCGCAGGA-3'	668

### CD4 genotyping by PCR-restriction fragment length polymorphism (PCR-RFLP) method

The PCR-RFLP technique in association with the restriction enzyme *Bse*RI was used to identify and differentiate between the two CD4 alleles. PCR amplification was performed on genomic DNA to amplify CD4 exon 3, and the PCR products were digested with an enzyme, *Bse*RI (New England Biolabs Inc., Ipswich, MA). The allele-specific bands were analyzed by 2 % agarose gel electrophoresis.

We determined the hereditary pattern of CD4 alleles of sibs from heterozygous parents by using the PCR-RFLP method to genotype 64 piglets, 35 males and 29 females, born from 17 matings. In addition, CD4 genotypes and phenotypes were assigned in 143 Microminipigs by the PCR-RFLP methods and flow cytometry as described above. The percentage and MFI of CD4+ cells in PBMCs were also compared between the two CD4 genotypes.

### Detection of CD4 mRNA by analyzing RT-PCR products

Peripheral blood samples of Microminipigs were collected into Paxgene Blood RNA tubes (PreAnalytiX, Hombrechtikon, Switzerland). Total RNA was extracted using a PAXgene Blood RNA Kit (PreAnalytiX). Complementary DNA (cDNA) was synthesized from oligo dT primers using total RNA and the reverse transcriptase kit, ReverTra Ace (TOYOBO, Osaka, Japan).

To characterise the expressed CD4 mRNA, RT-PCR amplification was performed between exon 1 and 4 or exon 1 and 5 by using two specific primer pairs (Table 2) that were designed from the nucleotide sequence information of two CD4 alleles, *CD4.A* and *CD4.B*, obtained from Microminpigs and the swine CD4 reference sequence [GenBank: NM_001001908]. RT-PCR was performed using the same conditions as those used for sequencing amplification. The RT-PCR products were digested with *Bse*RI, and electrophoresed in 2 % agarose gel.

**Table 2:** Primers for RT-PCR to detect the expression of CD4 mRNA

Name	Forward primer sequence	Reverse primer sequence	Length of PCR products (bp)
Primer 1	5'-GTAAGAGAAGCAGAGGGGAAGAG-3'	5'-GATTCTTGATGATCAGGGGAAAG-3'	400
Primer 2	5'-GTAAGAGAAGCAGAGGGGAAGAG-3'	5'-CATTCTTGCTTTTATTCCCTGGAC-3'	595

### Transfection of two kinds of CD4 alleles into HeLa cells

We chose HeLa cells for the analysis of CD4 expression analysis in Microminipigs because the same cells have been used for the analysis in human [12, 13]. To verify the differences in the antibody reactivity of CD4 alleles, HeLa cells were transfected with the FLAG-tagged CD4 vectors (CD4-FLAG) constructed by adding FLAG to C-terminus of the two different CD4 alleles. PBMCs were isolated from two pigs genotyped to two types of CD4 homozygotes using the Lymphoprep kit (Axis Shield) and their total RNAs were extracted with ISOGEN (NIPPON GENE, Tokyo, Japan). cDNA was synthesized by SuperScript III First-Strand Synthesis System (Life Technologies) using a sequence specific reverse primer (5′-TCAGGTGAGGGAATAGTTCTTCTGTTGCCG-3′). RT-PCR was performed with PrimeSTAR Max DNA Polymerase (TaKaRa Bio Inc., Otsu, Japan) and a CD4 specific primer pair containing *Xho*I and *Not*I recognition sequences and FLAG sequences (Forward: 5′-TATCTCGAGATGGACCCAGGAACCTCTCT-3′; Reverse: 5′- TATGCGGCCGCTCACTTGTCATCGTCCTTGTAATCGGTGAGGGAATAGTTCTTCTTCTGTTGC-3′). The RT-PCR products of CD4-FLAG were cloned into the mammalian expression vector pCAGGS [14], and the integrity of the constructed vectors were confirmed by DNA sequencing using the following five primers; Forward 1: 5′-GCAGGGACTTCCTTTGTCCCAAAT-3′; Forward 2: 5′- TATCTCGAGATGGACCCAGGAACCTCTCT-3′; Forward 3: 5′-AGTCACCCTACAGTGCAATGGAAAG-3′; Reverse 1: 5′- TATGCGGCCGCTCACTTGTCATCGTCCTTGTAATCGGTGAGGGAATAGTTCTTCTTCTGTTGC-3′; Reverse 2: 5′-TGTCCTTCCGAGTGAGAGACACAA-3′. The constructed plasmids were transfected into HeLa cells using Lipofectamine 2000 (Life Technologies) according to the manufacturer’s instructions. After culturing for 20 h, the transfected HeLa cells were stained with a rabbit anti-FLAG polyclonal antibody (Sigma-Aldrich, St. Louis, MO) followed by the Alexa Fluor 488 goat anti-rabbit IgG antibody (Life Technologies). The PE-conjugated mouse anti-pig CD4a monoclonal antibody (clone 74-12-4, SouthernBiotech, Birmingham, UK) was used for the CD4 molecule detection. The cells were also stained with Hoechst 33342 (ImmunoChemistry Technologies, Bloomington, IN). Fluorescence imaging was conducted using scanning laser confocal microscope FV1000-D IX81 (Olympus, Tokyo, Japan).

### Statistical analysis

In the hereditary analysis, the observed and theoretical values were assessed by the chi-squared test using Excel 2007 (Microsoft, Seattle, WA) with an add-in software Statcel 3 (OMS, Tokorozawa, Japan). Theoretical values were determined on the basis of the Punnett square. A difference of *P* < 0.05 was considered as significant.

## Results

PCR amplification of CD4 gene sequences between exons 2 and 10 was performed on 11 Microminipigs, six pigs that were CD4 antibody reactive and five pigs that were CD4 antibody unreactive. DNA sequencing and analysis of the 11 PCR products identified three homozygous and three heterozygous allelic sequences from the six CD4 antibody reactive samples, and five homozygous allelic sequences from the five CD4 antibody unreactive samples. We aligned the eight homozygous allelic sequences against the swine CD4 reference sequence [GenBank: NM_001001908] and identified two distinct allelic nucleotide sequences between exons 2 and 10 of the CD4 genes that we classified as alleles *CD4.A* and *CD4.B* (Additional file 1). In comparison with the [GenBank: NM_001001908] sequence, the *CD4.A* [DDBJ: LC064059] and *CD4.B* [DDBJ: LC064060] alleles had 15 and 22 nucleotide substitutions between exon 2 and 10 regions, respectively (Table 3). Nucleotide sequences identical to *CD4.A* have not been found in GenBank, and so far appear to be unique to the Microminipigs. In contrast, the nucleotide sequences of *CD4.B* were identical to that of the partial *CD4.2* sequence that reported only exons 3 and 4 in the CD4-undetectable NIH miniature swine [GenBank: X65630] [11].

**Table 3:** The number of nucleotide substitutions in *CD4.A* and *CD4.B* CDS compared to [GenBank: NM_00100908]

Allele	Exon	2	3	4	5	6	7	8	9	10
	The number of nucleotide substitutions									
*CD4.A*		0	1	1	2	3	3	5	1	0
*CD4.B*		0	12	1	1	3	0	5	1	0

**Fig. 1:**
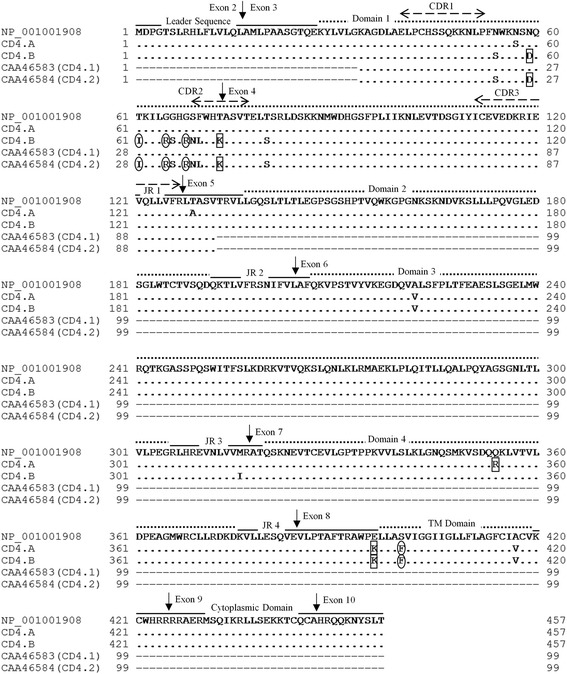
Comparison of amino-acid sequences of porcine CD4 alleles. Deduced amino-acid sequences of CD4.A and CD4.B were compared with those of the swine CD4 reference sequence [GenBank: NP_001001908]. Amino-acid sequences of two CD4 alleles reported in NIH miniature swine [GenBank: CAA46583 (CD4.1), CAA46584 (CD4.2)] also were compared with CD4.A and CD4.B. (-) indicates gaps or absence of sequence corresponding to [GenBank: NP_001001908]. (.) indicates having identical sequence with that of [GenBank: NP_001001908]. Arrow indicates the putative boundary of each exon. Regions of leader sequence, four extracellular Ig-like domains (Domain 1-4), joining region (JR 1-4), transmembrane (TM) domain, and cytoplasmic domain are also shown. <−−− > indicates CDR1-3 like region in domain 1. An outlined circle indicates that the amino-acid side chain has changed the polarity in association with amino-acid substitution. An outlined square indicates that the amino-acid side chain has changed the charge in association with amino-acid substitution

In comparing the derived CD4 protein sequences with the swine CD4 amino-acid reference sequence [GenBank: NP_001001908], the CD4.A and CD4.B protein sequences had seven and 15 amino-acid substitutions, respectively, in the regions of exons 2 to 10 (Fig. 1, Table 4). In CD4.A, there was one amino-acid substitution in three of the four extracellular domains as well as in the joining regions 1 and 4, and two amino-acid substitutions in the transmembrane domain. In CD4.B, there were ten amino-acid substitutions in domain 1, one in domain 3, one each in joining regions 3 and 4, and two in the transmembrane domain, some of which may change the polarity or charge of the amino-acid side chains. There was no amino-acid substitution in the cytoplasmic region of either CD4.A or CD4.B.

**Table 4:** The number of amino-acid substitutions in CD4.A and CD4.B compared to [GenBank: NP_001001908]

CD4 type	Regions of CD4 molecule									
	D 1	JR 1	D 2	JR 2	D 3	JR 3	D 4	JR 4	TM	CP
	The number of amino-acid substitutions									
CD4.A	1	1	0	0	1	0	1	1	2	0
CD4.B	10	0	0	0	1	1	0	1	2	0

D 1–4: domain 1–4; JR 1–4: joining region 1–4; *TM* transmembrane domain; *CP* cytoplasmic domain

**Fig. 2:**
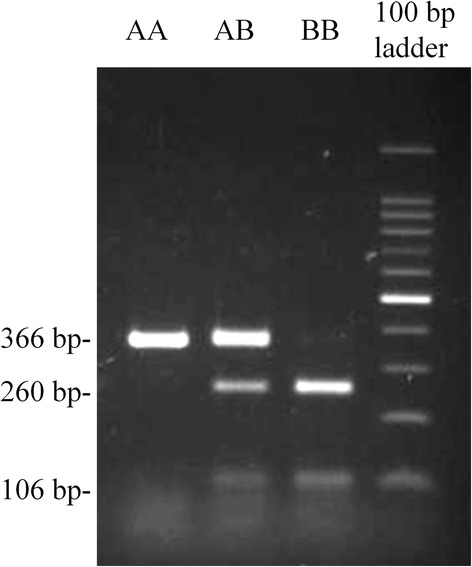
Electrophoretic pattern of PCR-RFLP of genomic DNA. The lanes are AA: *CD4.AA;* AB: *CD4.AB*; BB: *CD4.BB;* and the 100 bp ladder. The 366 bp-fragment was amplified from genomic DNA using primer pair for exon 3 (See Table 1). The PCR product was digested with *Bse*RI. PCR fragments with genotype of *CD4.AA*, *CD4.AB*, and *CD4.BB* showed single fragment (366 bp), three fragments (366, 260, and 106 bp), and two fragments (260 and 106 bp), respectively

**Fig. 3:**
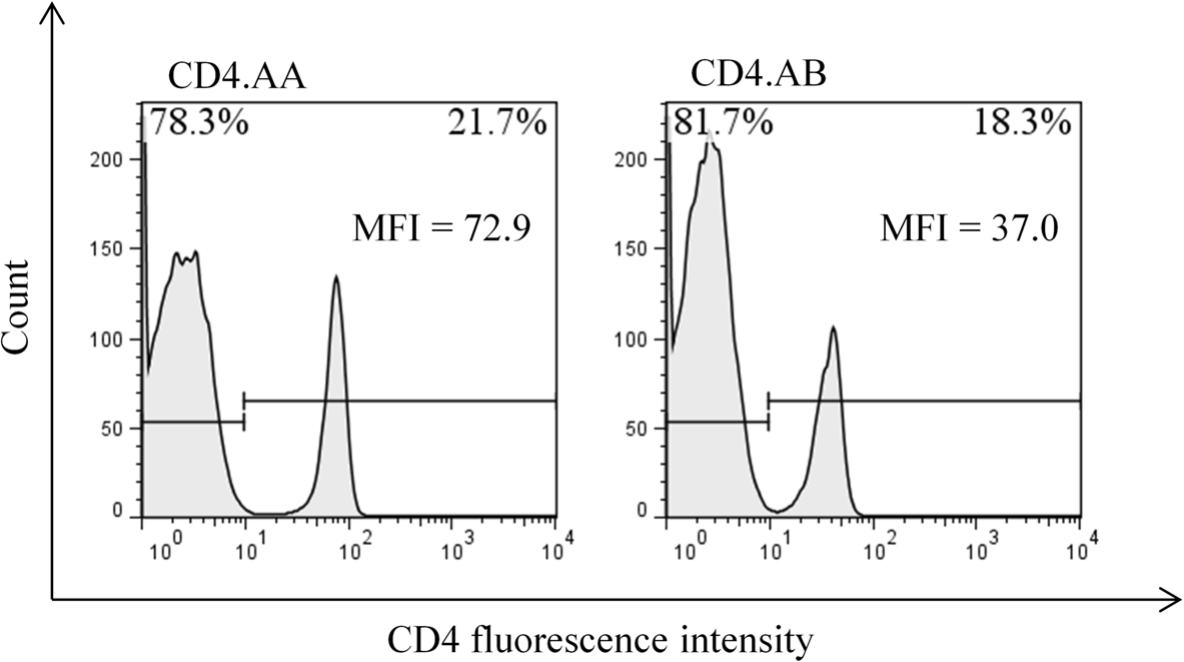
The percentage and MFI of CD4+ cells in PBMCs with *CD4.AA* and *CD4.AB*. CD4 genotypes in 2-month old Microminipigs assigned by PCR-RFLP. PBMCs were stained with FITC-conjugated anti-pig CD4 antibody and assessed by flow cytometry as described in Materials and Methods. The MFI of CD4+ cells with *CD4.AB* was almost half of those with *CD4.AA,* even though there was no significant difference in the percentage of CD4+ cells between *CD4.AA* and *CD4.AB*

Three CD4 genotypes in Microminipig herd were assigned as *CD4.AA*, *AB*, and *BB* by the PCR-RFLP method using *Bse*RI (Fig. 2). The restriction enzyme patterns of *CD4.AA*, *AB* and *BB* showed a single band (366 bp), three bands (366, 260, and 106 bp), and two bands (260 and 106 bp), respectively. The matings of 17 pairs of heterozygous parents revealed that the inheritance pattern of CD4 genotypes was autosomal (Table 5). As shown with the flow cytometry results in Table 6, PBMCs with *CD4.AA* and *AB* reacted with the antibody clone 74-12-4. In contrast, PBMCs with *CD4.BB* were unreactive with the antibody. The MFI of *CD4.AB* was approximately half the intensity of *CD4.AA*, even though the percentage of CD4+ cells in PBMCs were not different between the two CD4 alleles (Fig. 3).

**Table 5:** CD4 genotypes of piglets delivered from the matings of CD4 heterozygous pigs

CD4 genotype	The number of piglets		Theoretical value	
	Male	Female	Male	Female
*AA*	10	9	8	8
*AB*	16	13	16	16
*BB*	9	7	8	8
Total	35	29	32	32

The hereditary pattern corresponds to the theoretical value on the basis of autosomal heredity in heterozygous by analysis of chi-squared test (*P* > 0.05). Hence, the hereditary pattern of CD4 genotypes is autosomal in mode

**Table 6:** The relationship between CD4 genotype and affinity to anti-pig CD4 antibody

CD4 genotype	Number	CD4 reactivity	Number
*AA* *AB*	24 71	Reactive	95
*BB*	48	Non-reactive	48

CD4 genotype was assigned by PCR-RFLP method using *Bse*RI. CD4 reactivity was veriified by flow cytometry. CD4+ cells in PBMCs could not be detected with the antibody when they carried the *CD4.BB* genotype

**Fig. 4:**
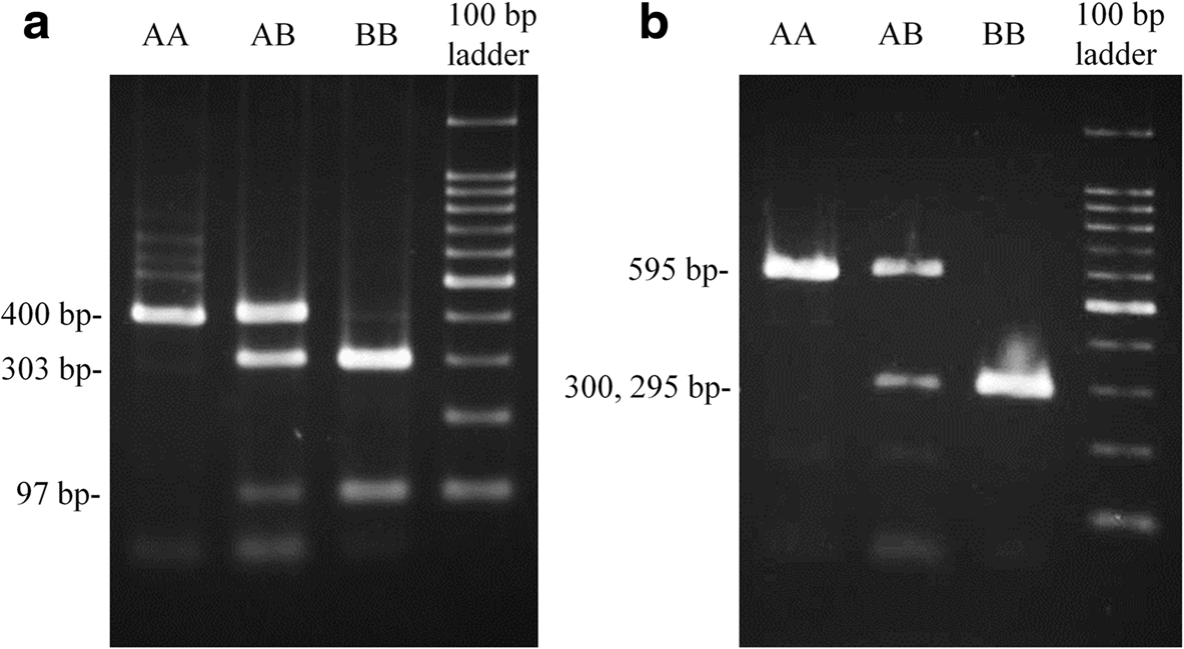
Electrophoretic pattern of RT-PCR products after enzyme digestion with *Bse*RI. The lanes are AA: *CD4.AA*; AB: *CD4.AB*; BB: *CD4.BB;* and the 100 bp ladder*.* The 400 bp (**a**) and 595 bp (**b**) of the CD4 sequence were amplified from cDNA using primer sets shown in Table 2 and the amplified products were digested with *Bse*RI. **a** After digestion with *Bse*RI, *CD4.AA*, *CD4.AB*, and *CD4.BB* showed a 400 bp-fragment (400 bp), three fragments of 400, 303 and 97 bp, and two fragments of 303 and 97 bp, respectively. **b** After digestion with *Bse*RI, *CD4.AA*, *CD4.AB*, and *CD4.BB* showed a 595 bp-fragment, three fragments of 595, 300 and 295 bp, and two fragments of 300 and 295 bp, respectively

CD4 gene expression was analyzed by RFLP using the RT-PCR sequence products that were amplified between exons 1 and 4 (Fig. 4a) or between exons 1 and 5 (Fig. 4b). RFLP distinguished the genotypes *CD4.AA*, *CD4.BB* and *CD4.AB* in both cases. In Fig. 4a, the RT-PCR products were detected as a single 400 bp-band by electrophoresis. After *Bse*RI digestion, the single band remained in *CD4AA*, whereas two digested bands of 303 bp and 97 bp were obtained in *CD4.BB*. Combinatorial band patterns of *CD4.AA* and *CD4.BB* were observed in *CD4.A/B*. In Fig. 4b, the undigested RT-PCR products were detected as a single 595 bp band. After the *Bse*RI digestion, the single 595 bp band remained in *CD4.AA*, whereas two digested bands of 300 bp and 295 bp were obtained in *CD4.BB*, and the combinatorial band patterns of *CD4.AA* and *CD4BB* were observed in *CD4.A/B*. Consequently, these results suggested that PBMCs with heterozygous CD4 genotype coexpressed *CD4.A* and *CD4.B* alleles at the mRNA level.

**Fig. 5:**
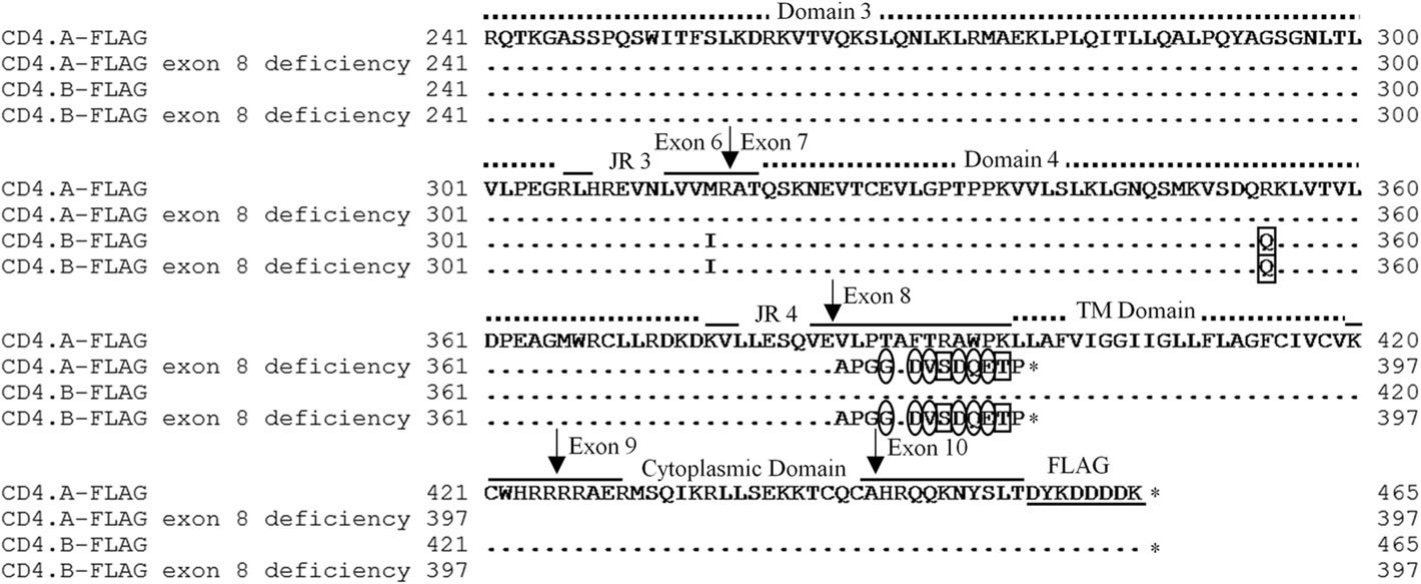
Alignment of amino-acid sequences of CD4.A-FLAG and CD4.B-FLAG and their exon 8 deficiency forms. (.) indicates having identical sequence to CD4.A-FLAG. Arrow indicates the putative boundary of each exon. (*) indicates the stop codon. The regions of two extracellular Ig-like domains (Domain 3, 4), joining region (JR 3, 4), transmembrane (TM) domain, cytoplasmic domain and FLAG are also shown. Exon 8 deficiency forms gave rise to a stop codon at the N-terminus of TM domain as a result of a frameshift from the beginning of the exon 8 region, even though amino-acid sequences of their external CD4 domains in the spliced forms were identical to those of CD4.A-FLAG and CD4.B-FLAG. Thus, we used the constructs with complete sequences of CD4.A-FLAG and CD4.B-FLAG for the expression study in HeLa cells

In validating the expression vector sequences, the insertion sequences of CD4.A-FLAG and CD4.B-FLAG were found to be identical to the genomic exon sequences described above (Additional file 1) except for the added FLAG sequence. Moreover, we also found a spliced form that lacked the CD4 exon 8 in both of the two CD4 alleles. These spliced forms with the exon 8 deficiency gave rise to a stop codon at the N-terminus of transmembrane domain as a result of a frameshift from the beginning of the exon 8 region, whereas amino-acid sequences of the external domains in the spliced forms were identical to those of the CD4.A and CD4.B derived from the nucleotide sequencing using genomic DNA (Fig. 5). Therefore, we used the constructs with complete sequences of CD4-FLAG for expression in HeLa cells. These alternative spliced forms were submitted to DDBJ (http://www.ddbj.nig.ac.jp) as *CD4.A exon 8 deficiency* [DDBJ: LC064061] and *CD4.B exon 8 deficiency* [DDBJ: LC064062].

**Fig. 6:**
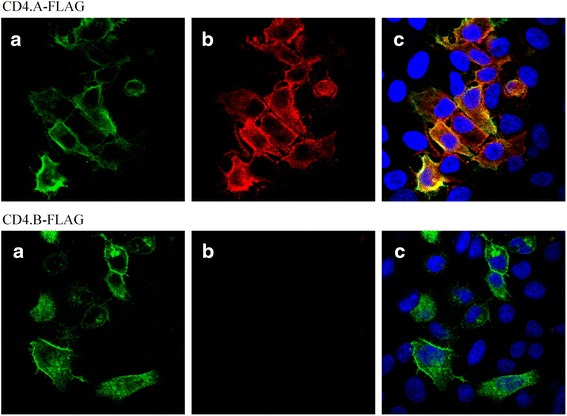
Expression of the CD4-FLAG vectors in HeLa cells. We expressed only the constructs CD4-FLAG with complete sequences in HeLa cells as described in Fig. 5. **a** Cells stained with rabbit anti-FLAG antibody followed by Alexa Fluor 488 goat anti-rabbit IgG antibody. **b** Cells stained with PE conjugated mouse anti-pig CD4 antibody. **c** Cells stained with the overlay of three fluorophore signals; anti-FLAG antibody, anti-pig CD4 antibody, and Hoechst 33342

Figure 6 shows the transient expression of CD4-FLAG without the exon 8 deficiency in HeLa cells. The CD4.A and FLAG proteins in CD4.A-FLAG were detected with the anti-pig CD4 antibody and anti-FLAG antibody. In contrast, the CD4.B in CD4.B-FLAG was unreactive with the anti-pig CD4 antibody even though FLAG was detected with the anti-FLAG antibody. These results show that we expressed the CD4.B protein in HeLa cells, but that we could not detect it with the anti-CD4 antibody.

## Discussion

The CD4 exonic sequences of the Microminipig CD4 gene were analyzed using the DNA isolated from CD4+ cells that were CD4-reactive and non-reactive to the 74-12-4 antibody, and two corresponding alleles, *CD4.A* and *CD4.B*, respectively, were identified. Although the CD4 gene is thought to be highly conserved, CD4 polymorphisms were reported previously in human, bovine, and ovine [15–17]. Also, two CD4 partial allelic sequences were reported in NIH miniature swine (*CD4.1*: X65629; *CD4.2*: X65630) [11]. The nucleotide sequences of exons 3 and 4 of *CD4.B* in Microminipigs were identical to those of *CD4.2* in NIH miniature swine. We could not conclude that *CD4.B* is identical to the complete CD4 gene sequence of NIH miniature swine because only the nucleotides of exons 3 and 4 were sequenced for *CD4.2*. However, the Microminipig breed is the result of crosses with western breeds, but it has no consanguinity with the NIH miniature swine [7]. Although the *CD4.B* of Microminipigs and *CD4.2* of NIH miniature swine might have a co-ancestor, the origins of *CD4.B* and *CD4.2* remain uncertain.

In comparing the amino-acid sequence alignments of CD4.A and CD4.B with the swine CD4 amino-acid reference sequence [GenBank: NP_001001908], we identified one amino-acid substitution in domain 4 of the extracellular region of CD4.A that involve alterations in the charge of the amino-acid side chain, and consequently a structural change in this domain of CD4. Domains 3 and 4 of CD4 play an important role in interacting with the T cell receptor-CD3 complex to influence signal transduction in T cell activation and function [18]. Therefore, the amino-acid substitution on domain 4 of CD4.A might affect signal transduction and T cell function during the interaction of CD4 with the T cell receptor-CD3 complex. The five amino-acid substitutions observed in domain 1 of CD4.B also alter the polarity or charge of amino-acid side chains and might elicit a structural alteration in this domain of the CD4. The domain 1 of the CD4 combines with the monomorphic region of the MHC molecule in the presentation of antigenic determinants to activate selected lymphocytes [3, 4]. Moreover, the amino-acid substitutions in the region of domain 1 in CD4.B correspond to the CDR2-like region of CD4.2 in NIH miniature swine and the CDR2-like region of CD4 in humans that bind to MHC class II molecules and the HIV envelope glycoprotein gp120 [11]. No clinical abnormalities have been observed as yet with the *CD4.B* genotype in Microminipigs, although the affinity of CD4.B to MHC class II might be different from that of CD4.A [9]. In this regard, the PCR-RFLP technique using *Bse*RI has allowed us to identify the three CD4 genotypes *CD4.AA*, *AB* and *BB* that correlated with CD4 reactivity to anti-pig CD4 antibodies. This simple CD4 genotyping method might be useful for selectively breeding *CD4.A* or *CD4.B* homozygous pigs and for developing association studies of immunity to infections and immunologically-related diseases.

Our PCR-RFLP study has demonstrated that both *CD4.A* and *CD4.B* are co-expressed in PBMC of heterozygous pigs. Moreover, the study on the expression of CD4-FLAG in HeLa cells confirmed that the amino-acid substitutions in CD4.B were associated with the loss of affinity to anti-CD4 antibody. On the other hand, the seven amino-acid substitutions observed within CD4.A reacted with the anti-CD4 antibody and one or other of them are likely to be linear or sequential epitopes recognized by the anti-CD4 antibody. Therefore, one or more of the ten amino-acid substitutions in domain 1 and/or the one amino-acid substitution in joining region 3 of CD4.B, which are not found in CD4.A, may have replaced the antigenic determinant and caused the lose of affinity for the anti-pig CD4 antibodies.

The detection of reduced levels of MFI in CD4 cells with *CD4.AB* in the Microminipigs appears to be due to the anti-CD4 antibody reacting with CD4.A, but not with the CD4.B molecules, if both types of CD4 proteins are coexpressed on the surface of helper T cells. Moreover, in CD4-FLAG insertion sequencing, the *CD4.A* and *CD4.B* alleles also had exon 8 deficiency forms that lacked the subsequent of transmembrane domain in the deduced amino-acid sequences. Thus, if the CD4 transcripts are without a transmembrane domain then the translated proteins might be secreted into the serum rather than be bound within the cellular membrane. The alternative spliced forms of CD4 CDS in swine have not been reported previously even though there are such variants registered in GenBank [GenBank: XM_005652591, GenBank: XM_005652592, GenBank: KC333254, GenBank: AY515293]. However, the lack of CD4 transmembrane region was reported for a mutant mouse model that secreted soluble CD4 without expression of membrane-bound CD4 [19]. This mouse model was used to show that soluble CD4 impaired a delayed-type hypersensitivity response by inhibiting IFN-γ production, and prohibited over-activation of CD4+ T cells by competitive inhibition of the binding of CD4 on the T-cell surface to MHC class II [20]. So, if the exon 8 deficiency forms are translated to the protein, the secreted CD4 might be also associated with prohibiting over-activation of CD4+ T cells in swine. Thus, further studies are needed to elucidate the significance of the expression of CD4 exon 8 deletions.

In NIH miniature swine, the functional differences of CD4 between CD4.1 and CD4.2 were investigated, but no differences were detected in antibody production against staphylococcal nuclease immunization and in the allogeneic mixed lymphocyte reaction [21]. In this regard, additional studies will be needed in Microminipigs to elucidate the functional significance or immunological importance of polymorphisms of the CD4 gene including the possible alternative spliced forms of the expressed gene. Because the CD4 molecule interacts with the MHC class II complex in antigen recognition [3, 4] the polymorphisms of both CD4 and MHC class II will need to be considered in future studies. The Microminipigs with defined SLA haplotypes [8] could be a useful animal model for further research on the interaction between CD4 allomorphs and MHC molecules in disease, infection and transplantation studies.

## Conclusions

Two CD4 alleles, named as *CD4.A* and *CD4.B*, were identified in Microminipigs, and these two alleles were found to express an exon 8 deficiency, indicating the potential for alternative spliced forms. The loss of reactivity of antigenic epitopes to an anti-CD4 antibody probably resulted because of amino-acid substitutions in CD4.B. These CD4 polymorphisms could be genotyped and identified simply by the PCR-RFLP technique using *Bse*RI in Microminipigs.

## Additional file

Additional file 1: Alignment of CD4 CDS based on swine CD4 reference sequence [GenBank: NM_00100908]. Nucleotide sequences of *CD4.A* and *CD4.B* were aligned based on swine CD4 reference sequence [GenBank: NM_00100908], and two CD4 alleles reported in NIH miniature swine were also aligned [GenBank: X65629 (*CD4.1*), GenBank: X65630 (*CD4.2*)]. (−) indicates having no sequence corresponding to [GenBank: NM_00100908]. (.) indicates having identical sequence with that of [GenBank: NM_00100908]. An arrow indicates an exon boundary. (TIF 1208 kb)
