# Supplementary figures and images for: Identification and characterization of two CD4 alleles in Microminipigs
Source: BMC Vet Res. 2016 Oct 7;12:222. doi: 10.1186/s12917-016-0856-8 (PMC5055687; doi:10.1186/s12917-016-0856-8)

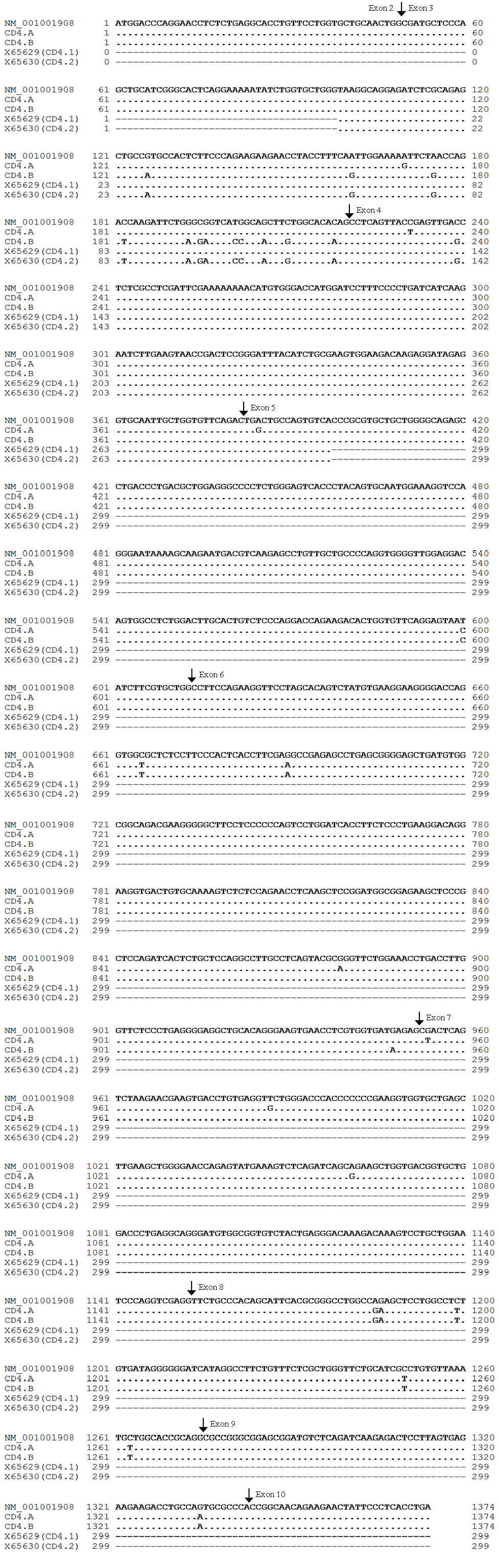

Supplement: Additional file 1: — Alignment of CD4 CDS based on swine CD4 reference sequence [GenBank: NM_00100908]. Nucleotide sequences of CD4.A and CD4.B were aligned based on swine CD4 reference sequence [GenBank: NM_00100908], and two CD4 alleles reported in NIH miniature swine were also aligned [GenBank: X65629 (CD4.1), GenBank: X65630 (CD4.2)]. (−) indicates having no sequence corresponding to [GenBank: NM_00100908]. (.) indicates having identical sequence with that of [GenBank: NM_00100908]. An arrow indicates an exon boundary. (TIF 1208 kb) [file 12917_2016_856_MOESM1_ESM.tif]
